# Compulsory Meat-inspection and Cattle-insurance in Saxony

**Published:** 1896-03

**Authors:** 


COMPULSORY MEAT-INSPECTION AND CATTLE-INSURANCE IN SAXONY. The Saxon Board of Agriculture emphasizes its complete agreement with the decision of the Chamber of Commerce in recommending plans for the introduction of a general and compulsory inspection of meat in connection with cattle insurance by the State, the Board of Agriculture to assume the cost of administering the laws and to pay an additional sum annually into the general treasury, because these regulations are only regarded as a support to other regulations which the Board has adopted at former sessions.—*Thierärzt Centralblatt*, December 1, 1895.

